# Editorial: Structural Studies of Protein Complexes in Signaling Pathways

**DOI:** 10.3389/fmolb.2021.641932

**Published:** 2021-04-30

**Authors:** Filippo Prischi, Panagis Filippakopoulos

**Affiliations:** ^1^School of Life Sciences, University of Essex, Colchester, United Kingdom; ^2^Structural Genomics Consortium, Oxford University, Oxford, United Kingdom

**Keywords:** signaling pathways, structural biology, protein structural and functional analysis, protein interaction network, proteins complexes, transmembrane proteins

Now, as never before in the history of humankind, people are constantly communicating and aware of their surroundings. It is astonishing to see how similar our society is to a basic cellular organism, a society of cells. In order to function and survive, cells need to communicate with each other and gather information about the external environment. They do not use mobiles or social media, but they are able to codify and transfer information using signaling pathways.

Signaling pathways are defined by an intricate network of protein-protein and protein-nucleic acid interactions that governs intra- and inter-cellular activities. Nearly all cellular processes are controlled by such interactions, including motility, growth, proliferation, gene expression, survival, and apoptosis. Structural biology has offered unprecedented insight into the function and regulation of protein-interaction networks. Advances at third generation synchrotron sources and the use of XFELs is having a massive impact on elucidating the structural rationale and dynamics of macromolecules (Moreno-Chicano et al., 2019). The technical advances in crystallography have also fueled developments in cryo-electron microscopy (cryo-EM), facilitating the determination of previously elusive protein structures at atomic or near-atomic resolutions. Recently, by optimizing the electron source for energy spread and reducing the noise by using a new energy filter and camera, the structures of two proteins, human GABA_A_R and mouse apoferritin, were solved at atomic resolution using cryo-EM, 1.7 and 1.2 Å, respectively (Nakane et al., 2020). At the other end of the structural biology spectrum there is NMR, historically considered highly complementary to crystallography. NMR presents major advantages in measuring the kinetic and thermodynamic parameters of a molecular system, and detecting and mapping transient or weak interactions. In their perspective article, Purslow et al. provide a technical overview of solvent Paramagnetic Relaxation Enhancement (solvent-PRE), Intermolecular Nuclear Overhauser Effect (NOE) and Residual Dipolar Coupling (RDC) for the study of protein complexes, and Chemical Shift Perturbation (CSP) for the determination of binding modes and K_D_ values for protein-ligand with weak interactions (K_D_ in the μM-mM range) in fast exchange regime (μs^−1^ NMR timescale). The authors also discuss several approaches to study medium size protein (~100 kDa) and overcome the molecular weight limit of solution NMR, focusing mostly on solid-state NMR sequences, like REDOR and PAINCP.

Signaling pathways are stimulated by the binding of extra- or intra-cellular signaling molecules to receptors, which relay signals via binding to downstream targets. The flow of information through the cell is mediated by specific, selective interactions, which determine the functional outcome of signaling networks. The resulting processes are tightly regulated and their misregulation has been associated with complex human pathologies, including cancer and neurodegenerative disorders. This Research Topic collects articles focusing on the use of structural biology and hybrid methods to characterize protein complexes and, most importantly, the formation of the correct protein complexes in response to specific signals.

Fenn et al. discussed the advances in the characterization of *Mycobacterium tuberculosis* Mce proteins, a class of membrane spanning proteins which promote bacterial survival within humans by manipulating host cell signaling. In a mini-review, the authors emphasized the roles of Mce beyond lipid transport and described the structural-functional features of three Mce complexes (Mce3E/Mce2E-ERK1/2, Mce2E-eEF1A1, and Mce3C-β2 integrin), highlighting the diverse mechanisms adopted by *M. tuberculosis* to downregulate cytokine expression and promote entry into macrophages.  Marshall and Bavro used a combination of structure-guided mutational studies to characterize another system linked to bacterial survival, the *Escherichia coli* tripartite efflux-pump AcrAB-TolC, assembly of which relies on the recognition and interaction of the outer-membrane factor TolC with the periplasmic adapter protein AcrA. Their results support the presence of a deep interpenetration of the AcrA-TolC interface and indicate that TolC contributes to the substrate selection during efflux events, uncovering an overlooked feature of the outer-membrane factor protein family.

Transmembrane proteins involved in cellular interactions or extracellular environment sensing are fascinating examples of highly spatial and temporal regulated signaling complexes.  Khan and Goult reviewed the diverse range of interactions triggered by integrin binding to talin and kindlin in the cytoplasm, and the extracellular matrix, highlighting the key role microscopy played in dissecting the organization of integrin adhesion complexes. The authors provide an elegant mechanosensitivity regulation model, describing extensively the link between auto-inhibition and the formation of inactive pre-complexes, further discussing the role autoinhibition plays in driving the correct protein interaction in response to a specific signal. Similarly, Guarino et al. provided a comprehensive review of the structural features that guide the clustering of acetylcholine receptors (AChRs), essential for the formation and maintenance of Neuromuscular junctions (NMJ) and synaptic connections. The authors critically discussed the well-characterized “canonical” (or agrin-induced) AChRs clustering model that involves interaction between the AChRs and the LRP4-MuSK complex bound to agrin, as well as the less well-characterized “non-canonical” model, exploring the possibility that the AChRs clustering is mediated by interactions with the Wnt-LRP4-MuSK complex. Chataigner et al. embarked on a challenging task to review the molecular mechanisms used by type-I transmembrane proteins to control adhesion and activation of signaling, focusing mostly on the extracellular portion of the proteins. Using different examples of prototypical transmembrane proteins, the authors focused on the roles X-Ray crystallography, NMR and cryo-EM played in unraveling proteins interactions, protein complexes structures and conformational changes underlying activation of signaling processes.

Despite the astonishing technical advances of the last 10–15 years in structural biology, experimental approaches are intrinsically time and resource demanding and computational studies, spanning from classic molecular dynamics simulations to more recent machine learning algorithms and network topology and proximity measures, have the potential to bypass some of these shortcomings. Zhang et al. present a quintessential application of computational study to explore the diverse functions of SMYD3 by analyzing its interactome. Using a pathway enrichment analysis coupled with structural analysis and sequence motif scanning, the authors identified two major classes of proteins forming transient interactions with SMYD3: epigenetic transcriptional regulators, like histone H3-4, and proteins related to calcium dependent signaling, such as PLCB3, supporting the hypothesis that SMYD3 is not merely a methyltransferase involved in histones modification, but could be an integral part of calcium signaling pathways.

The studies collected in this Research Topic represent just the tip of the iceberg of the possibilities offered by structural biology applications in understanding and characterizing signaling pathways, helping define future direction of research in biology and related fields. As the structural biology technologies and resources continue to improve, it will be possible to address additional biological challenges. We envision that in the next 10 years, a thorough structural understanding of larger and more complex systems will be possible, offering possibilities to map the dynamic processes in signaling networks in order to better understand the transmission of signals and the roles that different structural components have in relaying those signals within an organism.

## Author Contributions

All authors listed have made a substantial, direct and intellectual contribution to the work, and approved it for publication.

## Conflict of Interest

The authors declare that the research was conducted in the absence of any commercial or financial relationships that could be construed as a potential conflict of interest.
